# VEGF with AMD3100 endogenously mobilizes mesenchymal stem cells and improves fracture healing

**DOI:** 10.1002/jor.24164

**Published:** 2018-11-30

**Authors:** Richard Meeson, Anita Sanghani‐Keri, Melanie Coathup, Gordon Blunn

**Affiliations:** ^1^ Division of Surgery University College London Stanmore United Kingdom; ^2^ Royal Veterinary College Hertfordshire United Kingdom; ^3^ University of Central Florida Florida; ^4^ University of Portsmouth Portsmouth United Kingdom

**Keywords:** mobilization, mesenchymal stem cells, AMD3100, VEGF, fracture healing

## Abstract

A significant number of fractures develop non‐union. Mesenchymal stem cell (MSC) therapy may be beneficial, however, this requires cell acquisition, culture and delivery. Endogenous mobilization of stem cells offers a non‐invasive alternative. The hypothesis was administration of VEGF and the CXCR4 antagonist AMD3100 would increase the circulating pool of available MSCs and improve fracture healing. Ex‐breeder female wistar rats received VEGF followed by AMD3100, or sham PBS. Blood prepared for culture and colonies were counted. P3 cells were analyzed by flow cytometry, bi‐differentiation. The effect of mobilization on fracture healing was evaluated with 1.5 mm femoral osteotomy stabilized with an external fixator in 12–14 week old female Wistars. The mobilized group had significantly greater number of cfus/ml compared to controls, *p* = 0.029. The isolated cells expressed 1.8% CD34, 35% CD45, 61% CD29, 78% CD90, and differentiated into osteoblasts but not into adipocytes. The fracture gap in animals treated with VEGF and AMD3100 showed increased bone volume; 5.22 ± 1.7 µm^3^ and trabecular thickness 0.05 ± 0.01 µm compared with control animals (4.3 ± 3.1 µm^3^, 0.04 ± 0.01 µm, respectively). Radiographic scores quantifying fracture healing (RUST) showed that the animals in the mobilization group had a higher healing score compared to controls (9.6 vs. 7.7). Histologically, mobilization resulted in significantly lower group variability in bone formation (*p* = 0.032) and greater amounts of bone and less fibrous tissue than the control group. Clinical significance: This pre‐clinical study demonstrates a beneficial effect of endogenous MSC mobilization on fracture healing, which may have translation potential to prevent or treat clinical fractures at risk of delayed or non‐union fractures. © 2018 The Authors. *Journal of Orthopaedic Research*® Published by Wiley Periodicals, Inc. on behalf of Orthopaedic Research Society. J Orthop Res 37:1294–1302, 2019.

A significant number of bone defects and fractures do not heal. In the USA, it is estimated that around 100,000 fractures per year go on to non‐union.^1^ The UK National Health Service reports around 10% of fractures fail to heal, and treatment can be difficult requiring repeat surgery with a cost of up to £80,000 per patient.^2^ Successful fracture healing is reliant upon the recruitment, migration and homing of cells for inflammation, blood vessel formation, chondrogensis, osteogenesis.^3^ Adult mammalian bone marrow has two constituent stem cell populations with separate lineages, including haematopoietic stem cells which are non‐plastic adherent and will circulate in the peripheral blood, and stromal cells, which are plastic adherent, with a fibroblastic morphology and considered non‐circulating.^4^ Cells isolated from stroma have been shown in vitro and in vivo transplantation to be able to produce all tissues require for the bone organ^4^, ^5^ and are termed mesenchymal stem cells (MSCs). There is evidence for low numbers (1 in 10^6^, ^7^, ^8^ of nucleated cells) of peripheral blood circulating plastic adherent, osteogenic potent cells in mice, rabbits, guinea pigs and humans.^6^, ^7^ Clinical evidence from fracture patients also supports a role for circulating MSCs in fracture healing.^8^

The chemokine stromal cell derived factor 1 (SDF1, also known as CXCL12), and its receptor CXCR4, has a key role in stem cell migration from the bone marrow stroma into the circulation and is believed to be important for homing of stem cells to a fracture sites.^9^ Local increases in SDF1 expression have been measured in distraction osteogenesis, stress fractures and segmental defects.^9^, ^10^ It is suggested that when a fracture occurs there is a chemotactic gradient, with high levels of SFD1 at the fracture site, and subsequently increased levels in the blood steam, facilitating stem cell migration from their niches.^9^

The SDF1‐CXCR4 axis maintains HSCs and likely other stem cells in the bone marrow^9^, ^11^ and probably within other body niches. An intentional forced egress of cell ‘mobilization’ has been in clinical use for some time with haematopoietic stem cells for repopulation of the bone marrow after treatment for certain blood related malignancies.^12^, ^13^ AMD3100, (1,1‐[1,4‐Phenylenebis(methylene)] bis‐1,4,8,11‐tetraazacyclotetradecane octahydrochloride), is a bicyclam derivative that antagonises the CXCR4 receptor directly and mobilizes a population of CD34+ haematopoietic stem cells into the peripheral circulation. This occurs by highly selective, high affinity competitive blocking of the CXCR4 receptor which displaces stem cells from the bone marrow niche by disruption of their attraction to SDF1.^14^ Although haematopoietic stem cells have been successfully mobilized using GCSF or AMD3100 or a combination, work on MSC and other progenitors is limited and these cells do not seem to be as migratory as hematopoietic stem cells, potentially due to their larger size, increased niche adherence and their limited number in the bone marrow stroma.^15^ Pitchford's seminal work on different mobilization protocols in mice, demonstrated that AMD3100 combined with VEGF rather than GCSF, preferentially mobilizes a population of MSCs rather than haematopoietic stem cells.^11^

The aim of this study was to investigate whether administration of VEGF with AMD3100, could mobilize MSCs into the peripheral circulation of rats, and to determine whether increasing the circulating levels of MSCs would improve fracture healing in a delayed union rat femoral fracture model.

## METHODS

### Isolation of Bone Marrow MSCs (BMMSCs)

Healthy, ex‐breeder female wistar rats (*n* = 3) (450–550 g), were the donors. All procedures were carried out according to the UK Home Office Animals Scientific Procedures Act of 1986 and were approved by the Animal Welfare Ethical Review Board at the Royal Veterinary College, and aligned to the ARRIVE guidelines. The rats were euthanized and the femur aseptically isolated. The femoral medullary canal was flushed with 5 ml DMEM 4500 mg/L glucose, (Sigma–Aldrich, UK) with 20% fetal calf serum (FCS) and 1% penicillin/streptomycin (termed “media”), into a 25 cm^2^ polystyrene cell culture flask (Corning). The cells were cultured in a humidified incubator at 37°C, 95% air and 5% CO_2_. The media was changed after 5–7 days to remove non‐adherent cells and every 3–4 days thereafter. Once they had reached 70–80% confluence, they were passaged.

### Preparation of Growth Factors for Mobilization

Rat Vascular Endothelial Growth Factor 165 (VEGF) (PeproTech, 400–31) was prepared by dissolving the lyophilized product in sterile water to make a 0.1 mg/ml stock solution. A working solution was prepared by adding 1 ml of stock solution to 4mls of sterile PBS + 0.1%BSA (Sigma–Aldrich, UK), to achieve a 100 µg/ml injectable solution which was then aliquoted and stored at −20°C until needed. AMD3100 (Sigma–Aldrich A5602), stock solution was prepared by dissolving 5 mg lyophilized product in 0.5 ml sterile water, and then added to 4.5 mls PBS to produce a 1 mg/ml injection solution, which was then aliquoted and stored at −20°C, until needed.

### Mobilization of Peripheral Blood MSCs (PBMSCs)

For the mobilization study, the VEGF‐AMD group (*n* = 8) and PBS treated controls (*n* = 6) were healthy, ex‐breeder female Wistar rats (380–600 g). Rats were pre‐treated with VEGF (Peprotech), at 100 µg/kg, once daily by intra‐peritoneal (i.p.) injection daily for four days, at a volume of 0.5 mls/100 g. On day five, rats received a single i.p. 5 mg/kg dose of AMD3100, at a volume of 0.5mls/100g. The dosages of VEGF and AMD3100 were taken from Pitchford's et al.^11^ based on prior pharmacokinetic work^12^ and were adjusted to be appropriate for each individual rat's bodyweight.

**Figure 1:**
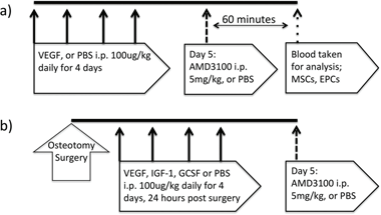
Experimental design and dosing schedule. Peripheral blood MSC mobilisation study (a). Endogenous enhancement of fracture healing study (b).

One hour post administration, rats were anaesthetized for terminal cardiac venipuncture. Controls were treated with PBS i.p. at the same volume and time intervals (Fig. 1a). Isolation of stem cells was achieved by red blood cell lysis where 10 ml of lysis solution (Red Blood Cell lysing Buffer Hybri‐Max solution Sigma–Aldrich, UK), was added per 1 ml of blood, and mixed in a 50 ml Falcon tube (Corning). After 5 min, 35 ml of PBS was added to neutralize the lysis solution, and then centrifuged at 400*g* for 5 min. The supernatant was aspirated and the cells were re‐suspended in media and plated into 25cm^2^ polystyrene cell culture flasks for MSC culture. The media was changed after five to seven days to remove non‐adherent cells and thereafter every 3–4 days. Colonies were counted at ×4 magnification placed under a phase‐contrast light microscope, using a grid overlay. Final CFU count was performed at 20 ± 2 days. Cells were passaged when they were 70–80% confluent, as previously described.

### Analysis of Isolated Cells

P3 cells, 30,000 cells per group were fixed and assessed using a combination of positive CD90 (Anti‐Mouse/Rat CD90.1 (Thy‐1.1) eBiosceince, UK) and CD29 (Anti‐Mouse/Rat CD29 (Integrin beta 1) eBioscience, UK), and negative MSC markers CD45 (Anti‐Rat CD45 eBioscience, UK) and CD34 (Anti‐CD34 abcam, UK) and were compared with appropriate isotopye controls. Analysis was performed using a flow cytometer (Cytoflex, Beckman Coulter, UK), with Cytexpert (Beckman Coulter, UK) software.

For differentiation, cells isolated from blood and from bone marrow were assessed by taking 30,000 P3 cells were seeded into a sterile 48 well plates, in triplicate and assessed for osteogenic and adipogenic differentiation through media supplementation for 21 days in comparison to standard media. Osteogenic media consisted of standard media with 100 nM dexamethasone (Sigma–Aldrich), 50 μg/ml l‐ascorbic acid 2‐phosphate (Sigma–Aldrich) and 10 mM Glycerol‐2‐phosphate disodium salt hydrate, (Sigma–Aldrich). Adipogenic media consisted of standard media with 0.1 mM dexamethasone (Sigma–Aldrich), 0.45 mM IBMX (Sigma–Aldrich), 10 mg/ml Insulin (1.7 mmol/L) (Sigma–Aldrich), and 50 mM Indomethacin (Sigma–Aldrich). Osteogenic differentiation was assessed by staining with Alizarin red stain (0.2 Molar, pH 4.32, Sigma–Aldrich), to identify calcium mineral deposition. Adipogenic differentiation was assessed with Oil red O staining for lipid droplets.

### Femoral Delayed Union Fracture Model

For the fracture model, 12–14 week old female Wistar rats were assigned to the control (*n* = 7) or mobilization group (*n* = 8) (230–300 g). According to the Home Office license and under aseptic conditions, a lateral approach was made to the left femur. Using anatomical landmarks, and a custom precision jig‐guide system, four bicortical threaded 1.4 mm stainless‐steel fixator pins were consistently placed in the craniomedial femur. Pins were exited through separate stab incisions and the custom variable spacing fixator was attached. A mid‐diaphyseal femoral osteotomy, with no periosteal stripping was made using a diamond tipped hand‐saw, while applying sterile saline coolant/lubricant. A precision spacer ensured a fixed distance between the cis cortex and connecting blocks of 9 mm. The fixator was then attached and the osteotomy gap distracted to 1.5 mm using a second precision spacer. The biceps femoris was closed over the osteotomy with a single horizontal mattress suture (1.5M PDS II, Ethicon, UK), and then the skin was closed with intradermal continuous suture (1.5M monocryl, Ethicon, UK). Activity was unrestricted post surgery. Twenty‐four hours post‐surgery, rats were given a single i.p. injection of either VEGF (100 μg/kg), or PBS once daily for four days. On day five, they were given a single injection of AMD3100 (5 mg/kg). All i.p. injections including AMD3100 and sham PBS were administered at a volume of 0.5mls/100g bodyweight based on the day 0 pre‐surgical weight (Fig. 1b). All procedures were carried out at the Royal Veterinary College, North Mymms, in accordance with the Animals Scientific Procedures Act 1986, and aligned to the ARRIVE guidelines. Those taking part in any surgical procedure held UK Home Office licences. The dosages of VEGF and AMD3100 were taken from Pitchford et al.^11^ and the timing of mobilisation relative to surgery was based on prior work.^16^, ^17^

After five weeks, the left femur including the fixator, were retrieved from the sacrificed rats. Femurs were fixed and scanned with a Bruker Skyscan 1172 micro‐tomograph machine (Bruker, Belgium), at 60KV, 167uA with a 0.5 mm aluminum filter. A rotation step of 0.5°, without frame averaging, and an image pixel size of 4.89 µm. MicroCT scans were reconstructed using NRecon (Bruker, Belgium). Analysis was performed using CTAn (Bruker, Belgium). The central 60% of the osteotomy gap was assessed (0.9 mm = 180 slices at 5 µm thick). Radiographic scouts were assessed for bone union, using orthogonal projections isolated from the microCT image acquisition series. Radiographs were evaluated by three independent assessors in a randomized and blinded manner and graded according to the RUST scoring.^18^

After microCT analysis, the bones were decalcified in ethylenediaminetetraacetic acid (EDTA, Sigma–Aldrich), sequentially dehydrated in alcohol solutions, de‐fatted and embedded into wax with the fixator pins orthogonal to the facing surface of the block. Fixator blocks and pins were removed once the wax had set and a sledge microtome (ThermoFisher Scientific, UK) was used to make 5 μm thick slices. The alignment of the blocks within the microtome was altered as necessary to ensure a central sagittal slice through the femur, assessed using the fixator pin tract holes. Slides were prepared and stained with Haematoxylin and Eosin (Sigma–Aldrich). Histomorphometric analyses were performed at 2× magnification, quantified with a 1.5 mm scaled width line‐intercept grid, with 120 intersections; grid squares were 160 µm in both directions. Intersections were then scored as bone, cartilage, fibrous tissue, vascular or void.

### Statistical Analysis

Due to the relatively small group sizes (*n* < 9), non‐parametric tests were performed to compare groups including Mann–Whitney U (MWU). Assessment of data spread was made with a Levene's test for equality of variance. Significance was set a *p *< 0.05 and tests were analyzed with SPSS version 24 (IBM, Chicago). Data are expressed as mean ± SD unless otherwise specified.

## RESULTS

### Comparison of BMMSCs to PBMSCs

BMMSCs formed fibroblastic colonies (CFUs) with spindle shaped cells. P3 cells were able to differentiation down osteogenic and adipogenic lines, with positive staining with Alizarin red and Oil red O, respectively. BMMSCs had mean expression levels of the following markers: CD45 5.7 ± 5.0%, CD34 0.2 ± 0.1%, CD29 98.4 ± 4.1%, and CD90 98.9 ± 1.0%.

**Figure 2:**
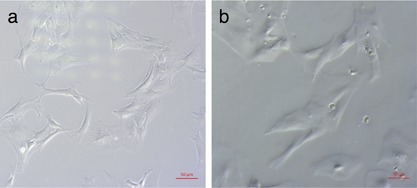
Light microscopy image (×10 magnification) of third passage bone marrow derived MSCs at day seven (a), compared third passage day seven peripheral blood MSCs mobilized with VEGF and AMD3100 (b). Bone marrow MSCs were obtained from the femoral shaft of rats of a similar age and isolated by plastic adherence. Peripheral blood MSCs were obtained by cardiac puncture 60 min after a single dose of AMD3100, preceded by a 4 day course of VEGF, once daily, every 24 h. The cells were isolated by plastic adherence after lysing the red blood cells.

No CFUs were isolated from the non‐mobilized control blood. The mobilized group formed CFU‐Fs in six out of the eight individual cultures. Initially, round mononuclear cells were present with some immature elongating cells. Over time, the cells began to take on a similar morphology to those obtained by bone marrow isolation; spindle or fibroblastic‐shaped cells, with a centrifugal arrangement of cells to form a colony. Notably, however, they took longer to form clear CFUs, typically around 14–18 days, when compared with bone marrow isolated cells (5–7 days). The mean CFU‐F/ml for the mobilized group was significantly higher at 2.9 ± 1.8 CFU‐F/ml (*p* = 0.029) than the controls. These cells were passaged up to P3 (Fig. 2a shows bone marrow derived and 2b shows blood mobilised MSCs).

Flow cytometry of P3 cells showed cells were CD34 negative, however, CD45 which is typically negative in MSCs, was positive on some cells with both CD45 positive and CD45 negative cells present, varying from 9% to 60% positive within any population. MSC markers CD90 and CD29 were relatively highly expressed at 78% and 64%, respectively (See Table 1 for details).

**Table 1:** Flow Cytometry Analysis of Cell Surface Marker Expression of VEGF AMD3100 Mobilized PBMSCs

					CD34−	CD34+	CD45+	CD45+
Rat	CD45+	CD34+	CD29+	CD90+	CD45−	CD45−	CD34−	CD34+
1	60.9	3.0	76.2	77.8	37.0	2.6	59.3	1.0
2	35.4	1.8	60.7	83.9	61.3	2.2	36.1	0.4
3	8.6	1.8	55.2	70.7	87.0	2.3	10.6	0.2
Mean	35.0 ± 26.2	2.2 ± 0.7	64.0 ± 10.9	77.5 ± 6.6	61.8 ± 25.0	2.4 ± 0.2	35.3 ± 24.2	0.5 ± 0.4

The three right hand columns show the co‐expression of CD markers. In cells from all the rats there is low expression of CD34 but in 2 of the animals investigated there is a relatively high co‐expression of CD45, which is usually not found on MSCs. There is high expression of CD29 and CD90, which are known MSC markers.

**Figure 3:**
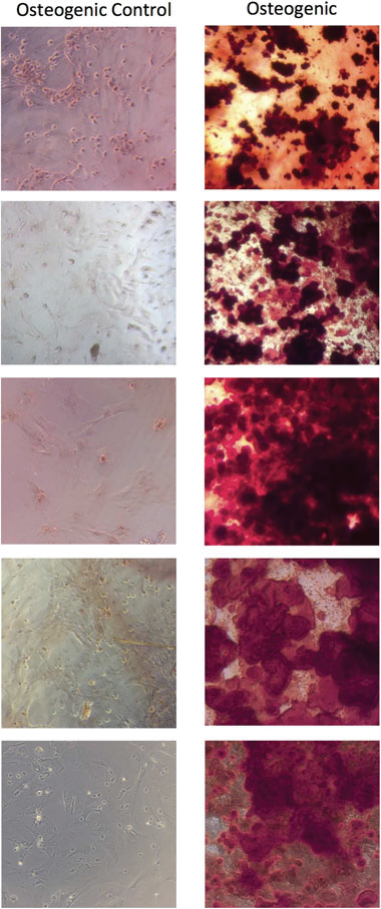
Light microscopy images (×10 magnification) of third passage cells from peripheral blood MSCs in rats treated with VEGF and AMD3100. The cells were cultured with osteogenic supplements for 21 days and stained with Alizarin red demonstrating mineral formation. Each row represents a culture from a different rat.

In differentiation assays, mobilized P3 PBMSCs formed a monolayer within seven days. During supplementation with osteogenic media, the cell morphology became less spindaloid and more cuboidal and multiple small granules became apparent. Staining for Alizarin red after 21 days demonstrated red stained calcium deposits in all samples, consistent with osteoblast activity (*n* = 5) (see Fig. 3), however despite adipogenic supplementation there was no evidence of adipogenic differentiation, with no positive Oil Red O staining evident in 4/5 and one showing a very small amount. This was in contrast to the cells isolated directly from the bone marrow, which demonstrated mineralisation and lipid production when differentiated down the respective lineages.

### Influence of Mobilization on Fracture Healing

**Figure 4:**
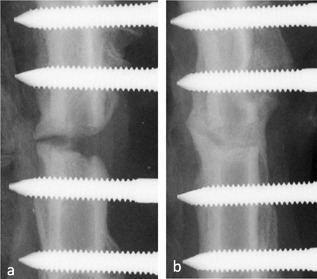
MicroCT scout radiographs, showing an example of the 1.5 mm gap control group (a) and the VEGF AMD3100 treated group (b) taken after 5 weeks This shows a non‐union at 5 weeks in the control animal with union and bone formation in the gap in the treated animal.

RUST scoring gave a mean of 7.71 ± 2.7 for the control group and 9.63 ± 1.3 for the VEGF‐AMD group (Fig. 4 shows a low scoring [a] and high scoring [b] example). On microCT analysis, mobilized group (*n* = 8), showed a higher mean bone volume than controls (5.22 ± 1.7 µm^3^ vs. 4.3 ± 3.1 µm^3^), as well as increased trabecular thickness (0.048 ± 0.007 µm vs. 0.042 ± 0.003 µm). The overall data spread was significantly reduced in the VEGF‐AMD3100 group when looking at tissue volume (TV µm^3^, *p* = 0.036) and trabecular number (*p* = 0.048) (Table 2).

**Table 2:** MicroCT Quantitative Morphometry Indices of Bone Formation Within the 60% of the Osteotomy Gap Where TV (µm^3^) = Tissue Volume, BV (µm^3^) = Bone Volume, TV/BV (%) = Percentage Bone Volume, TS (µm^2^) = Tissue Surface, BS (µm^2^) = Bone Surface, Tb.Th (µm) = Trabecular Thickness, Tb.Sp (µm) = Trabecular Separation, Tb.N (1/µm) = Trabecular Number

MicroCT Parameter	1.5 mm Control	VEGF‐AMD
TV (µm^3^)	9.23 ± 6.14	10.03 ± 3.22
BV (µm^3^)	4.31 ± 3.08	5.22 ± 1.71
TV/BV (%)	53.79 ± 20.82	52.52 ± 5.85
TS (µm^2^)	62.83 ± 45.55	63.56 ± 19.88
BS (µm^2^)	326.15 ± 220.05	355.52 ± 130.15
Tb.Th (µm)	0.04 ± 0.01	0.05 ± 0.01
Tb.Sp (µm)	0.07 ± 0.03	0.08 ± 0.02
Tb.N (1/µm)	14.09 ± 9.32	10.99 ± 1.08
Total Porosity (%)	46.21 ± 20.82	47.48 ± 5.85

MicroCT analysis of the mobilized group showed a higher mean bone volume than controls as well as increased trabecular thickness. The overall data spread was analyzed using a Levene's test for equality of variance and found to be significantly reduced in VEGF‐AMD3100 group for tissue volume (TV µm^3^, *p* = 0.036) and trabecular number (*p* = 0.048), suggesting less variation in degree of healing between individuals.

**Figure 5:**
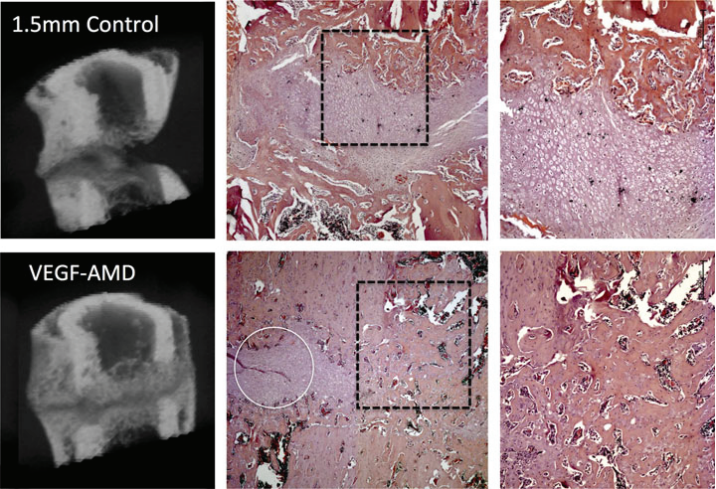
MicroCT 3D reconstructions of mid femoral regions, with a mid‐sagittal reveal showing the associated mid sagittal histology section, stained with hematoxylin and eosin, centered on the osteotomy at ×2.5 magnification, and then enhanced region at ×5 magnification. An example from a non‐treated control animal is shown in the upper images and a VEGF/AMD3100 treated animal in the lower images. The microCT in control groups show limited union with a large gap, which has not been filled‐in with bone. In the control animal there is also evidence of cortical bone resorption although a periosteal callus has formed. The microCT from the VEGF‐AMD3100 group shows almost complete bone union in the periosteal callus, and incomplete union in the endosteal callus, with the osteotomy filled with mostly mineralized tissue. Histology shows that the tissue in the gap of the control animal is composed of cartilage while cartilage is present in the gap in the treated animal there are regions where bone bridges between the fracture ends have formed. The white line encircles a small region of remaining cartilage within the osteotomy in the treated animal.

Histomorphometric analysis revealed proportionally greater levels of bone in the osteotomy of the mobilized group; 55.1 ± 7.8% bone, 40.9 ± 9.8% cartilage, 0.3 ± 0.8% fibrous, and 3.9 ± 3.9% vascular tissue (Fig. 5). The combined bone and cartilage percentage was 96 ± 3.7. This compared with control osteotomy tissue composition of 39.1 ± 23.9% bone, 43.1 ± 24.6% cartilage, 15.3 ± 37.4% fibrous and 2.4 ± 2.0% vascular tissue; and the combined bone and cartilage within the gap was 82.3 ± 36.4%. Levene's test for equality of variances showed a significant decrease in the variability of healing in the mobilized group for bone (*p* = 0.032) and fibrous tissue (*p* = 0.026).

## DISCUSSION

This study has demonstrated a significant increase in peripheral blood circulating PBMSCs post VEGF and AMD3100 administration. Notably, they were morphologically similar to BMMSCs, but were osteoblastic lineage limited, and had lower levels of MSC markers (CD90, and CD29) and higher levels of CD45, likely indicating a heterogeneous population of adherent fibroblastic expandable cells. Critically, the mobilization of this population with VEGF and AMD3100 was associated with an improvement in fracture healing. Mobilized fractures had increased bone volume and thicker struts of woven bone on micro CT analysis, near doubling of union rates, with a significant reduction in data variation for the treated group on bone volume from microCT data, and a smaller standard deviation with the RUST radiographic score, with improved means. This suggests mobilization may preferentially have influence on the poorest healing individuals, which may be clinically beneficial. Histology corroborated the findings on microCT with an increase in bone volume, vascularization and reduced fibrous tissue within the osteotomy.

Under the in vitro protocol, no PBMSCs were isolated from the non‐mobilized blood samples. Others have had more success,^19^ however, they used younger animals. The isolation of cells was performed in ex‐breeders, which are adult through to geriatric, and age related changes in stem cell differentiation, proliferation and metabolism^20^ are reported. Older rats have fewer CFUs, fewer MSCs in the bone marrow,^21^ possibly explaining the reduced yields in non‐mobilized controls and mobilized groups compared with Pitchford et al.,^11^ who used young mice. Humans also show reduced CFUs with ageing^6^ and a recent study also showed an age relationship with CXCR4 dependent migration^22^ and hence the effect of antagonism of the CXCR4‐SDF1 axis may also reduce with age. Most of the studies looking at peripheral blood circulating stem cells and mobilization in animal models have used young immature animals,^11^, ^17^, ^19^, ^23^ and therefore there may be merit in evaluating the role of age in mobilization potential. Although the rats were not ovariectomized, assessing mobilization in ex‐breeders is more relevant, as the likely target population for translation will be fractures in older patients and in those with osteoporosis where fracture healing is more likely to be impaired than in young individuals.

It is assumed that the mobilized cells are of bone marrow origin, however, with systemic administration of the mobilizing agents, they may be from other niches, particularly when considering the conservation of SDF1‐CXCR4 axis goes beyond the bone marrow niche.^10^, ^24^, ^25^ It is certainly suggested that the bone marrow makes a contribution to the mobilized peripherally circulating cells, as demonstrated by isolating the femoral artery and vein^11^ to evaluate mobilization from an isolated body compartment. The authors suggest that the cells are therefore bone marrow in origin, however an isolated femoral component actually only informs of all possible niches within the hind limb, and not exclusively the bone marrow compartment. It remains possible that these cells were mobilized from sources other than the bone marrow, such as the periosteum, muscle or perivascular niches.^26^ Interestingly the mobilized cells did not have adipogenic potential, but had effective osteoblastic differentiation, which may be important for bone tissue engineering. The traditional view is that a single MSC would be capable of differentiation into osteoblasts, chondrocytes or adipocytes. Even classical bone marrow stem cell work has shown different clonal populations frequently do not have full tri‐differentiation, while interestingly, the osteogenic lineage is always present.^27^ Other studies have suggested a hierarchy of sequential lineage loss with the potential for osteogenic differentiation remaining.^28^

They showed that only 1/3 clones were capable of tri‐differentiation, and the majority were osteo or chondro orientated. Due to the paucity of cells and the long duration of culture required with PBMSCs, there were insufficient cells for pellet culture and chondrogenic differentiation, and therefore it remains unclear if the PBMSCs could also be differentiated into chondrocytes. The PBMSCs mobilized had similar cell surface marker expression as the BMMSCs, although CD29 and CD90 were not as highly expressed. Notably there appeared to be the presence of two CD45 populations; CD45− and CD45+ groups, both without CD34 expression. CD45 is a key leukocyte marker and MSCs are universally considered to be CD45‐29, 30; however, CD45 has been demonstrated on cultured bone marrow MSCs from patients with haematological malignancies. These cells could differentiate and were morphologically similar to CD45‐ MSCs, and hence under certain circumstances the CD45 rule may be broken.^31^ It is impossible to tell if these cells were simply contaminants or did represent a CD45+ MSC population. Other studies in rabbits^32^ and a comprehensive comparison of rat bone marrow to blood MSCs showed no difference in CD marker expression, morphology and tri‐lineage potential, although growth and differentiation potential are reduced in peripheral blood isolated MSCs.^33^

Although VEGF with AMD3100 statistically increased the yields of PBMSCs, they were still relatively low. Conceptually, a single CFU is produced from a single stem/progenitor.^5^ In this model system, the average yield of three stem/progenitor cells per ml of blood combined with a maximum 6–10 ml available made evaluation challenging. To that end, in vitro identification with expansion was necessary, and allowed a wider platform of evaluation, although cell expansion was also slow. This methodology was similar to Pitchford's work in mice,^11^ however, they showed in young mice greater stem cell mobilization of 15 MSCs/ml blood. This finding, may be an artefact of in vitro culture and the processing performed to remove red blood cells prior to culture, or relate to species or age differences in the donors. The problems of isolating stem cells from the peripheral circulation is not new however, and other groups have used fibrin microbeads that bind matrix‐dependent cells to concentrate the proportion of MSCs within MNCs to improve subsequent plating density and yields.^34^ A further consideration is the single time point of sampling, which essentially provides only a “snap‐shot” of the circulating pool.

Evidently, the mobilization of a peripheral blood MSC like osteoblastic progenitor was beneficial in a compromised fracture healing environment. Previous work using a similar fixator system showed complete union at five weeks with a 0.5 mm osteotomy and an atrophic non‐union with a 3 mm osteotomy.^35^ A 1.5 mm osteotomy was chosen as a half‐way measure with the expectation of compromised healing as a good test base for the effect of mobilization on fracture healing. This study was the first study to evaluate the potential effects of stem‐progenitor mobilization on fracture healing in rats. Critically, it evaluated the potential to recue a compromised healing environment, rather than simply augmenting an uncomplicated healing situation.

Mobilization using VEGF with AMD3100, improved fracture healing, with an increased RUST score. The trabecular thickness and the space between trabecular (trabecular separation) evaluation output provides commentary on the nature of the woven bone formed within the osteotomy in this instance. The mobilized group had an increased the trabecular separation, implying of the bone formed was more porous, but increase trabecular thickness suggested the individual struts were thicker. Perhaps this represents a more advanced or more rapidly developed stage of endochondral ossification. In any case, VEGF with AMD3100 increased the bone formation over the controls. Others have seen improvements in fracture healing using AMD3100 alone,^17^ or combined with IGF1.^16^ These models were in young mice and showed improved healing, in a non‐compromised situation. There is therefore cross‐species merit in this strategy, and the ideal mobilization protocol remains unclear, particular in a compromised healing environment which may be a more appropriate test system considering the likely translation. Notably, on microCT and histomorphometric analysis, the variation in healing was significantly reduced with VEGF‐AMD3100 treatment, perhaps indicating it improved the poorer healing individuals more than the better healing ones, and thus decreased the variability of healing seen in the controls.

Clearly the nature of the disturbance to the SDF1‐CXCR4 axis is important. It is likely that a short duration blockade will mobilize more stem and progenitor cells and hence increase the total pool available to the fracture site. However, the homing to the fracture site also relies upon the very interaction being antagonized. The short half‐life of AMD3100,^14^ likely provides a “pulse” in the early inflammatory phase of fracture healing has more mobilizing effect at the bone marrow or other niches, rather than significantly impairing the recruitment of cells to the fracture site over days to weeks. Longer term blockade throughout the period of fracture healing will significantly reduce callus cartilage, callus size and bone formation, with reduced expression of genes associated with endochondral ossification.^10^ Continued AMD3100 administration also reduces new bone formation in distraction osteogenesis models,^36^ and therefore the timing of the administration of VEGF and AMD3100 relative to the stage of fracture healing is likely to be crucial and warrants further investigation.

A difference between the CFU mobilization analysis and the subsequent evaluation in fracture healing in this study is presence and influence of the osteotomy. It is well know that fractures release increased levels of growth factors.^37^ Clinically, VEGF has been shown to be increased in human patients with long bone fractures within the first couple of weeks, lasting up to six months post trauma.^38^ Conceivably the administration of VEGF which is thought to pre‐prime the bone marrow to preferentially release MSCs when ADM3100 is given,^11^ could have had direct humoral influence on the fracture healing itself, irrespective of stem cell mobilization. Histomorphometric assessment of vascularisation in the mobilized group showed higher levels of blood vessels, and VEGF is a known potent angiogenesis promoter with a clear role in endochondral and intramembranous bone formation.^39^, ^40^ Exogenous VEGF administration will inevitably have both direct and indirect effects on bone formation. VEGF can act indirectly through its receptors on endothelial cells, influencing the development of a new vascular network, allowing bone orientated stem and progenitors to migrate into the fracture callus and differentiate into osteoblasts.^41^

An important aspect of any evaluation of fracture healing is associated with the functional results. There have been a number of articles that assessed the strength of repair and the degree of bone formation. The majority of these show good correlation, however, this was not performed in this proof of concept study due to the increased numbers of rats that would have been required to achieve sufficient statistical power. Now that we have established efficacy, future dose regime evaluations would include this aspect.

In order to progress this study further it may be necessary to optimise the administration of both VEGF and AMD3100. In this instance we chose to administer the protocol immediately after fracture, during the early inflammatory phase of fracture healing, when stem cells will be recruited, and is consistent with other studies.^16^, ^17^ This is sensible based on the translational potential of this treatment as it would have to be given after a fracture occurred, but could be instigated early on as a prophylactic in “at risk” fracture patients such as fragility fractures. Nonetheless, the optimal timing of the administration of VEGF and AMD3100 relative to the stage of fracture healing could impact on its influence on fracture healing and warrants further investigation. Different dosing or timings, such as repeated injections of AMD3100, may increase the circulating pool of MSCs, however, for these cells to be effective they have to home to the site of fracture through the SDF1 CXCR4 interaction. Fortunately the half‐life of AMD3100 is short allowing the cells to re‐establish their homing capacity, and only studies that gave AMD3100 throughout the entire fracture healing process showed impaired healing.^10^

Future mechanistic work could include spiking the circulation with labeled stem cells at the same time that AMD3100 is administered, and then investigating the efficiency of these cells to migrate to the fracture suite over time. However, there is an issue of determining cell migration characteristics from cultured cells as cell surface protein expression critical for migration and homing are influenced by in vitro cell culture. Whether or not cultured labelled cells would reflect the in vivo kinetics of uncultured cell migration is unclear. Only bone marrow ablation with recapitulation with labelled marrow, or parabiotic studies could adequately answer this question and they are highly problematic to perform due to significant animal welfare implications.

There are other methods that may be used to enhance mobilisation of stem cells. For example Meng et al. transfected MSCs to express Aquaporin, a regulator of endothelial cell migration, and showed the cells had enhanced migration in a transwell assay.^42^ SOX 11 transfected stem cells transcriptionally activate Runx2 and CXCR4 expression, and when administered in a rat femoral fracture model they showed a larger number of MSCs migrated to the fracture site and improved bone fracture healing.^43^ While these studies show that migration of cells can be manipulated and can improve bone healing in fracture models, the advantage of our approach where the CXCR4/SDF1 axis is disrupted means that cells are not manipulated in vitro, and the approach is entirely endogenous.

Further work to determine the influence of age on mobilization, the role of other cells populations mobilized and optimization of both the in vitro assessment and the dose/timing of therapy, and combinations of other growth factors with AMD3100 should be pursued. In conclusion, VEGF combined with AMD3100 can mobilize a population of MSC‐like osteoblastic lineage determined cells into the peripheral circulation and this leads to increases in bone formation in a delay union osteotomy model. Notably, this approach shows promise in a challenging fracture‐healing environment, which differentiates this work from prior studies augmenting normal healing, and for the first time, a MSC mobilising protocol of VEGF‐AMD3100 has been evaluated. Clinically there could be significant benefit to fragility and other at risk fracture to provide a “biological boost” to the fracture healing process in at risk patients, through this non‐invasive endogenous strategy.

## AUTHORS' CONTRIBUTIONS

RM grant funding, experimental design, experimental work and manuscript preparation. AS experimental work and manuscript review. MC grant funding experimental design, manuscript review. GB grant funding, experimental design, experimental work, manuscript preparation. All authors have read and approved the final submission.
